# Quality of life and pruritus in patients with severe sepsis resuscitated with hydroxyethyl starch long-term follow-up of a randomised trial

**DOI:** 10.1186/cc12586

**Published:** 2013-02-25

**Authors:** Piotr Wittbrodt, Nicolai Haase, Dominika Butowska, Robert Winding, Jesper B Poulsen, Anders Perner

**Affiliations:** 1 Department of Anaesthesia and Intensive Care, Herning Hospital, Gl. Landevej 61, 7400 Herning, Denmark; 2 Department of Intensive Care, Rigshospitalet, Blegdamsvej 9, 2100 Copenhagen, Denmark; 3 Faculty of Health and Medical Sciences, University of Copenhagen, Blegdamsvej 3, 2100 Copenhagen, Denmark

## Abstract

**Introduction:**

The effects of hydroxyethyl starch (HES) on patient-centered outcome measures have not been fully described in patients with severe sepsis. We assessed health-related quality of life (HRQoL) and the occurrence of pruritus in patients with severe sepsis randomized to resuscitation with HES 130/0.42 or Ringer's acetate.

**Methods:**

We did post hoc analyses of the Danish survivors (*n *= 295) of the 6S trial using mailed questionnaires on self-perceived HRQoL (Short Form (SF) - 36) and pruritus.

**Results:**

Median 14 months (interquartile range 10 to 18) after randomization, 182 (61%) and 185 (62%) completed questionnaires were obtained for the assessment of HRQoL and pruritus, respectively. Responders were older than nonresponders, but characteristics at randomization of the responders in the HES vs. Ringer's groups were comparable. At follow-up, the patients in the HES group had lower mental component summary scores than those in the Ringer's group (median 45 (interquartile range 36 to 55) vs. 53 (39 to 60), *P *= 0.01). The group differences were mainly in the scales of vitality and mental health. There was no difference in the physical component summary scores between groups, but patients in the HES group scored worse in bodily pain. Forty-nine percent of patients allocated to HES had experienced pruritus at any time after ICU discharge compared to 43% of those allocated to Ringer's (relative risk 1.13, 95% confidence interval 0.83 to 1.55, *P *= 0.43).

**Conclusions:**

At long-term follow-up patients with severe sepsis assigned to resuscitation with HES 130/0.42 had worse self-perceived HRQoL than those assigned to Ringer's acetate whereas there were no statistically significant differences in the occurrence of pruritus.

## Introduction

Hydroxyethyl starch (HES) has been one of the most frequently used fluids for resuscitation in intensive care units (ICUs) worldwide [1]. However, recent trials of HES 130/0.38 to 0.45 (molecular weight/substitution grade) have shown increased frequency of adverse events and use of renal replacement therapy and blood products in general ICU patients in addition to increased mortality in patients with severe sepsis [2,3]. Moreover, HES treatment has been shown to cause pruritus described as dose-dependent, delayed in onset and persistent [4]. Studies report pruritus occurrence in none to 55% of patients [5-12] and it is controversial if HES 130/0.38 to 0.45 causes less pruritus than the HES solutions with higher molecular weight and substitution grade [10]. The effects of HES 130/0.38 to 0.45 on patient-centered outcome measures have not been fully described in patients with severe sepsis. The purpose of this study was to assess self-perceived health-related quality of life (HRQoL), being one of the most important indicators of health care [13] and the occurrence of pruritus in long-term survivors of the Scandinavian Starch for Severe Sepsis/Septic Shock (6S) trial [3]. We hypothesized, that the HES group would have worse HRQoL and pruritus than those in the Ringers group.

## Materials and methods

### Participants

The study population comprised the Danish survivors of the 6S trial as identified in the National Patient Registry. The 6S trial was investigator-initiated, multicenter, blinded, stratified, parallel-grouped using a computer-generated allocation sequence and centralized, blinded randomization. The inclusion and exclusion criteria are given in Additional file 1 and the CONSORT diagram of the screening, randomization and the 90-day follow-up process appears in the original publication and in the study protocol [3,14]. The participants in the 6S trial were randomly allocated to fluid resuscitation in the trial ICUs between December 2009 and November 2011 using either 6% HES 130/0.42 in Ringer's acetate (Tetraspan 6%, B Braun Medical, Melsungen, Germany) or Ringer's acetate (Sterofundin ISO, B Braun Medical). The unblinding of the trial occurred on 24 March 2012.

In March 2012, printed copies of the short form (SF)-36 and a self-composed pruritus questionnaire were sent to all the Danish survivors together with a letter explaining the purpose of the follow-up study. Nonresponders were contacted by telephone in May 2012. Consent was obtained at the inclusion in the 6S trial, and the post hoc analyses and renewed contact with the patients were approved by the Danish Medicines Agency and the Ethics Committee for the Capital Region of Denmark. At the time of contact, none of the patients knew the results of the 6S trial or which trial fluid they had received.

### Assessment of HRQoL

The SF-36 comprises three levels: items (questions), scales scoring the items and main summary scores aggregating the scales - the physical and mental component summary (PCS and MCS) scores. It is a self-administered questionnaire consisting of 36 items measuring eight scales of health: physical functioning (PF), role of limitations due to physical health (RP), bodily pain (BP), general health perceptions (GH), vitality (VT), social functioning (SF), role of limitations due to emotional problems (RE), and mental health (MH). Since its creation in the early 1990s the questionnaire has been used and validated in different clinical settings including ICU patients [15-17].

We used the Danish version of the questionnaire. To adapt the original (English, USA) version of SF-36, all the items have been translated as described by the International Quality of Life Assessment protocol [18] and validated in accordance with the local characteristics of the population [19,20].

### Assessment of pruritus

Pruritus was assessed by a self-composed questionnaire (see Additional file 1) including a visual analog scale (VAS) to describe the intensity of pruritus. We asked about the occurrence of pruritus in the past two days as well as at any time after the index ICU admission, actions taken to relieve pruritus, specifying the use of ointments, tablets and contact to general practitioner, dermatologist or other.

### Statistical analyses

The investigator (PW) analyzing the data was blinded to the assigned trial fluids. The predefined primary outcome measures were the PCS and MCS scores for the HRQoL part and any pruritus after ICU discharge ('Yes' in either question 1 or 2, see Additional file 1) for the pruritus part. The secondary outcome measures were the scale scores for the HRQoL part, pruritus in the last 48 hours, the severity of pruritus and actions taken against pruritus.

SF-36 was scored according to the manual [21]. Three different methods were applied to assess the influence of missing data on the results: 1) a complete-case analysis rejecting scales where *any *item was missing, 2) analyses of imputed data using missing data estimation (MDE) software provided by Quality Metrics Inc. (Lincoln, RI, USA) and 3) two best/worst-case analyses where missing answers in the HES group were set to the worst possible answer and missing answers in the Ringer's group were set to the best possible answer and vice versa. The latter analyses were done after unblinding of the investigator performing the analyses.

All the obtained scores for the eight scales and PCS and MCS scores were described as median values with interquartile ranges (IQR) and compared between the trial groups using the Mann-Whitney U test. The response consistency index (RCI) was calculated to assess the potential inconsistencies within the relevant items of the SF-36. The scales were evaluated for internal consistency by Cronbach's alpha coefficients and for the discriminate validity between the mental and the physical components.

To evaluate the occurrence of pruritus in total, we described the percentage of affirmative answers in questions 1 and 2 of the questionnaire, while the severity was assessed by the median scores on VAS and compared between the treatment groups using the chi-square test and Mann-Whitney U test, respectively. The answers to questions concerning actions taken against pruritus were described as percentages and compared between groups using chi-square testing. The statistical analyses were done using Health Outcomes Scoring Software (Quality Metrics Inc.), SPSS Statistics version 20.0 (IBM, Armonk, NY, USA) and SAS 9.2 (SAS Institute Inc., Cary, NC, USA). *P *values < 0.05 were considered significant.

## Results

**Figure 1:**
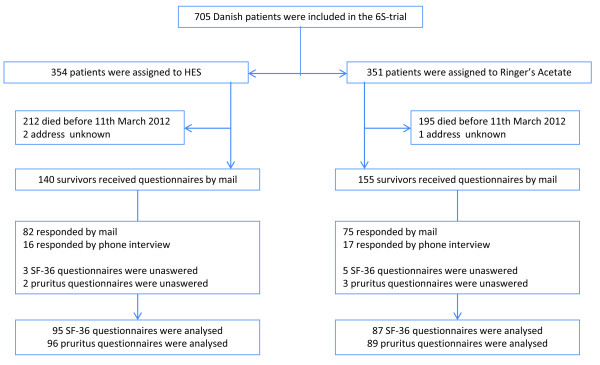
**Flowchart of the data collection process**. Reasons for nonresponding were as follows: eight were too sick to answer (four in each group), six were unwilling to participate and for the remaining ninety-one patients our attempts to reach them by telephone were unsuccessful.

The inclusion process yielded 182 (HRQoL) and 185 (pruritus) valid responses from a total of 295 different patients median 14 months (IQR 9 to 18) after randomization. The response rates were 61% for HRQoL and 62% for pruritus (Figure 1). The responders were older and more had shock at randomization compared to the nonresponders (Table 1). Among the responders the baseline characteristics at randomization and time to follow-up (Table 2) were comparable between patients assigned to HES and those assigned to Ringer's.

**Table 1:** Characteristics at randomization of responders and nonresponders.

	Responders (*n *= 190)	Nonresponders (*n *= 105)	*P *value
**Allocated to HES - n (%)**	98 (52)	42 (40)	0.06
**Age - years**	66 (59-74)	56 (49-66)	< 0.0001
**Male gender - no. (%)**	105 (55)	65 (62)	0.27
**Included at university hospital - no. (%)**	71 (37)	37 (35)	0.72
**Surgery^1 ^- no. (%)**			
Emergency	64 (34)	29 (28)	0.28
Elective	24 (13)	10 (10)	0.42
**Source of sepsis^2 ^- no. (%)**			
Lungs	80 (42)	59 (56)	-
Abdomen	73 (38)	24 (23)	-
Urinary tract	26 (14)	15 (14)	-
Soft tissue	23 (12)	15 (14)	-
Other	22 (12)	8 (8)	-
**SAPS II**	49 (38-58)	47 (34-55)	0.16
**SOFA score^3^**	7 (5-9)	7 (5-9)	0.64
**Shock^4 ^- no. (%)**	160 (84)	76 (72)	0.02
**Acute kidney injury^5 ^- no. (%)**	71 (37)	34 (32)	0.39
**Mechanical ventilation - no. (%)**	98 (52)	59 (56)	0.45
**Comorbidities**			
Diabetes mellitus - no. (%)	28 (15)	23 (22)	0.12
Arterial hypertension - no. (%)	81 (43)	33 (31)	0.06
Chronic renal disease^6 ^- no. (%)	23 (13)	20 (21)	0.11
Hematological malignancy - no. (%)	15 (8)	3 (3)	0.08
**Previous admission for - no. (%)**			
Heart failure or myocardial infarction	22 (12)	9 (9)	0.42
Stroke	13 (7)	11 (10)	0.27
Asthma or COPD	24 (13)	15 (14)	0.69

^1^Numbers are for patients who underwent surgery during the index hospitalization, but before randomization. ^2^Some patients had more than one source of infection. The 'other' category included sepsis from a vascular catheter-related infection, meningitis, or endocarditis, as well as sepsis from unknown sources. ^3^The SOFA scoring was modified because cerebral failure was not assessed. ^4^Shock at randomization was defined as a mean arterial pressure of less than 70 mm Hg, the need for ongoing treatment with vasopressor or inotropic agents, or a plasma lactate level of more than 4.0 mmol per liter in the hour before randomization. ^5^Acute kidney injury was defined as a renal SOFA score of 2 or higher (plasma creatinine level > 170 μmol/l (1.9 mg/dl) or urinary output < 500 ml/d). ^6^Chronic renal disease was defined as a preadmission plasma creatinine > 100 μmol/l (1.2 mg/dl). HES, hydroxyethyl starch; SAPS, simplified acute physiology score; SOFA, sequential organ failure assessment, COPD, chronic obstructive pulmonary disease.

**Table 2:** Characteristics at randomization of patients who responded to at least one of the questionnaires.

	HES 130/0.42 (*n *= 98)	Ringer's acetate (*n *= 92)
**Time to follow-up - months**	13 (9-17)	14 (10-18)
**Age - years**	66 (59-74)	66 (58-75)
**Male gender - no. (%)**	52 (53)	53 (58)
**Included at university hospital - no. (%)**	35 (36)	36 (39)
**Surgery^1 ^- no. (%)**		
Emergency	31 (32)	33 (36)
Elective	7 (7)	17 (18)
**Source of ICU admittance - no. (%)**		
Emergency department	32 (33)	21 (23)
General ward	40 (41)	39 (42)
Operating or recovery room	17 (17)	21 (23)
Other ICU in the same hospital	2 (2)	2 (2)
Other hospital	7 (7)	9 (10)
**Source of sepsis^2 ^- no. (%)**		
Lungs	42 (43)	38 (41)
Abdomen	40 (41)	33 (36)
Urinary tract	13 (13)	13 (14)
Soft tissue	9 (9)	14 (15)
Other	10 (10)	12 (13)
**Positive culture from blood or a sterile site - no. (%)**	17 (17)	11 (12)
**Time from ICU admission to randomization - hours**	3 (1-11)	3 (1-14)
**SAPS II**	48 (36-58)	50 (38-59)
**SOFA score^3^**	7 (5-9)	7 (5-9)
**Shock^4 ^- no. (%)**	80 (82)	80 (87)
**Acute kidney injury^5 ^- no. (%)**	36 (37)	35 (38)
**Mechanical ventilation - no. (%)**	44 (45)	54 (59)
**Comorbidities**		
Diabetes mellitus - no. (%)	12 (12)	16 (17)
Arterial hypertension - no. (%)	42 (43)	39 (42)
Chronic renal disease^6 ^- no. (%)	9 (10)	14 (17)
Hematological malignancy - no. (%)	6 (6)	9 (10)
**Previous admission for - no. (%)**		
Heart failure or myocardial infarction	10 (10)	12 (13)
Stroke	7 (7)	6 (7)
Asthma or COPD	12 (12)	12 (13)
**Any ventilation in the ICU, n (%)**	71 (72)	72 (78)
**Days in ventilator if treated, median (IQR)**	6 (3-14)	5 (3-12)
**Any renal replacement therapy dialysis in the ICU, n (%)**	18 (18)	15 (16)
**Days in renal replacement therapy if treated, median (IQR)**	7 (3-22)	10 (6-22)
**Days in ICU, median (IQR)**	6 (3-14)	8 (4-14)
**Days in hospital in the 90 days follow-up period, median (IQR)**	32 (20-50)	30 (20-53)

^1^Numbers are for patients who underwent surgery during the index hospitalization, but before randomization. ^2^Some patients had more than one source of infection. The 'other' category included sepsis from a vascular catheter-related infection, meningitis, or endocarditis, as well as sepsis from unknown sources. ^3^The SOFA scoring was modified because cerebral failure was not assessed. ^4^Shock at randomization was defined as a mean arterial pressure of less than 70 mm Hg, the need for ongoing treatment with vasopressor or inotropic agents, or a plasma lactate level of more than 4.0 mmol per liter in the hour before randomization. ^5^Acute kidney injury was defined as a renal SOFA score of 2 or higher (plasma creatinine level > 170 μmol/l (1.9 mg/dl) or urinary output < 500 ml/d). ^6^Chronic renal disease was defined as a preadmission plasma creatinine > 100 μmol/l (1.2 mg/dl). HES, hydroxyethyl starch; SAPS, simplified acute physiology score; SOFA, sequential organ failure assessment, COPD, chronic obstructive pulmonary disease; IQR, interquartile range.

### Data quality

Completeness of data in SF-36, defined as completed responses divided by the total possible number of responses was 96%. In the cases of twenty patients, it was impossible to calculate all the scales. All the obtained responses were within range for the individual items. Eighty-four percent of the responses were defined as consistent using RCI, with a total of 30 questionnaires containing items with sporadic inconsistencies (in one or two questions). We were able to score all the scales in 90% of the cases without using any imputation of missing data, which increased to 97% after imputation of missing data.

The internal consistency of the items, defined by correlation of 0.4 with their hypothesized scale was 100%. The item MH4 'have you felt downhearted and blue' correlated better (0.61) with VT than with its hypothesized scale MH. All the other items showed highest correlations with their designed scales. The reliability estimates, defining the correlation grade, (with the exception of PF) ranged from 0.83 (MH) to 0.89 (RP); PF reached the value of 0.94.

Most of the questionnaires evaluating pruritus were complete (0.5 to 11% missing answers), and there were no inconsistencies within the questionnaires.

### Health-related quality of life

The patients allocated to HES had significantly lower MCS score, bodily pain, vitality, social function and mental health than those allocated to Ringer's (Table 3). There were no statistical differences in PCS scores between the intervention groups. Comparable results were observed in the complete-case analyses with the exception of social function (*P *= 0.07 without imputation). The best/worst-case analyses did not change the direction of the intervention effect, but only mental health remained statistically different when the missing values in the HES group were substituted with the best possible score and those in the Ringer's group the worst possible score (see Table S1 in Additional file 1).

**Table 3:** Health-related quality of life in patients with severe sepsis allocated to HES 130/0

	Imputation dataset					Complete case dataset				
	
	HES group		Ringer's group			HES group		Ringer's group		
Scale	N	IQR	N	IQR	*P *value	N	IQR	N	IQR	*P *-value
**Primary outcomes**										
PCS	88	37 (29-48)	83	40 (32-51)	0.23	74	38 (29-48)	63	42 (33-52)	0.15
MCS	88	45 (36-55)	83	53 (39-60)	0.01	74	46 (35-56)	63	54 (41-59)	0.02
**Secondary outcomes**										
PF	94	50 (20-75)	86	65 (30-85)	0.17	91	50 (20-75)	81	65 (30-85)	0.23
RP	87	0 (0-75)	82	13 (0-75)	0.63	85	0 (0-75)	79	25 (0-75)	0.46
BP	95	52 (31-84)	86	73 (42-100)	0.007	91	52 (31-84)	83	72 (42-100)	0.02
GH	95	42 (30-62)	87	52 (25-72)	0.35	86	41 (30-62)	80	52 (30-72)	0.19
VT	94	45 (29-60)	85	55 (35-75)	0.008	92	45 (28-60)	82	55 (35-75)	0.004
SF	93	75 (38-100)	86	88 (63-100)	0.03	88	75 (38-100)	81	75 (63-100)	0.07
RE	87	33 (0-100)	81	67 (0-100)	0.19	84	33 (0-100)	78	67 (0-100)	0.18
MH	93	64 (52-82)	86	80 (60-92)	0.004	91	64 (52-84)	85	80 (60-92)	0.006

Data from the imputed and complete case datasets are given. Values are medians and interquartile ranges (IQR). *N *= number of patient where the scales could be calculated. HES, hydroxyethyl starch; PCS, physical component summary; MCS, mental component summary; PF, physical functioning; RP, role physical; BP, bodily pain; GH, general health; VT, vitality; SF, social functioning; RE, role emotional; MH, mental health.

### Pruritus

There were no statistical significant differences between the groups in the occurrence or severity of pruritus (Table 4). More patients in the HES group had used ointments or tablets against pruritus than those in the Ringer's group (Table 4). Seven patients (six in the Ringers group) indicated that they had pruritus in the last 72 hours prior to answering the questionnaire, but not before.

**Table 4:** Pruritus in patients with severe sepsis allocated to HES 130/0

	HES 130/0.42	Ringer's acetate	Relative risk (95%CI)	*P *value
**Primary outcome**				
Pruritus at any time after discharge, n/N (%)	47/96 (49)	38/88 (43)	1.13 (0.83-1.55)	0.43
**Secondary outcomes**				
Pruritus in the last 48 hours, n/N (%)	31/95 (33)	24/89 (27)	1.21 (0.77-1.89)	0.40
VAS score, median (IQR)*	4.5 (3.5-5.5)	3.5 (2.0-6.5)	-	0.28
Action taken against pruritus n/N (%)	36/96 (37)	20/88 (23)	1.65 (1.04-2.62)	0.03
Use of ointment/tablets n/N (%)	36/96 (37)	19/88 (21)	1.74 (1.08-2.79)	0.02
Consulted GP n/N (%)	11/96 (11)	7/88 (8)	1.26 (0.53-2.99)	0.60
Consulted dermatologist n/N (%)	2/96 (2)	5/88 (6)	0.37 (0.07-1.84)	0.20

*N *= number of patients in the treatment group. *n *= number of cases. *Medians are for those who had pruritus within the last 48 hours. HES, hydroxyethyl starch; VAS, visual analogue scale; IQR, interquartile range, GP, general practitioner.

## Discussion

In post hoc analyses of this multicenter, blinded, randomized trial of fluid resuscitation in severe sepsis, HRQoL at long-term follow-up was lower in patients allocated to HES 130/0.42 compared to those allocated to Ringer's acetate. The differences in HRQoL were observed in the MCS score, three scales describing the mental health and in one scale describing the physical health of the patients.

To the best of our knowledge this is the first report on HRQoL after fluid therapy with HES. We can only speculate on the mechanisms by which HES adversely affected HRQoL. As the patients allocated to HES had more bleeding episodes, higher use of blood products and renal replacement therapy and fewer days out of hospital in the 90-day trial period after randomization, all these factors may have contributed to lower HRQoL compared with patients in the Ringer's group [3]. In addition, long-term toxic effects of HES deposited in kidney, liver and bone marrow have been documented [22-25]. HES does not seem to cross the intact blood-brain barrier in animal models, however, it does accumulate in the peripheral nerves [26,27]. These toxic effects may affect the general health and thereby HRQoL of the patients, but we do not have data to substantiate such effects in our patients.

There were no statistically significant differences in the occurrence or severity of pruritus between the two intervention groups. The point estimates did favor the Ringer's group and more patients in the HES group had used medications against pruritus. That HES may cause pruritus also in ICU patients is supported by data from the CHEST trial, where HES 130/0.4 doubled the occurrence of pruritus compared to saline in general ICU patients [2]. It is noteworthy that a considerable number of patients in the Ringer's group reported pruritus in our study, suggesting that this is very frequent in survivors of severe sepsis. We observed many more patients with pruritus than in the CHEST and CRYSTMAS trials [2,5]. The reasons for these differences may be due to differences in the timing, methods/questionnaires used to capture pruritus and to the different HES doses and patient populations in these trials. There are no details on the methods for pruritus assessment in the reports of the CHEST and CRYSTMAS trials.

The PCS and MCS scores observed here are within the range of those observed in other studies of HRQoL using SF-36 in long-term survivors of sepsis [16,28]. More importantly, the PCS and MCS scores observed are well below those in the general population underlining the importance of HRQoL as an outcome measure in patients with severe sepsis in general and in trials in particular. The latter is substantiated by our observation of differences in HRQoL between patients in the two intervention groups.

There are several strengths to this study. The risk of bias was minimized by preparing the analysis plan before the analyses were done and both patients and the investigator analyzing the data were blinded to the intervention. Also the intervention groups were comparable at randomization. We used a validated tool to assess HRQoL, obtained good data quality and the results were comparable in the analyses of the imputed and complete-case datasets.

The study has several limitations. These were post hoc analyses of the Danish survivors only. We made contact with patients at the same time point, so that the follow-up time varied. Only 60% of the survivors responded and these patients were older and more had shock at baseline than the nonresponders. We cannot know if any of these limitations introduced bias. In 10% of the responses not all the scales were computable. As any missing data introduce uncertainty, we based our analyses on the currently recommended statistical approach [29], which is to impute data rather than to trust the complete-case analyses. The imputation lowered the number of cases with missing data to 3%, which likely minimized the potential bias. In addition, the best/worst-case analysis, which is the most conservative approach, did not change the direction of the intervention effect, but only the difference observed in MH remained significant when the missing data in the HES group were given the best possible score and those in the Ringer's group the worst possible score. It is unclear which magnitude of difference in SF-36 scores represents a meaningful difference for patients [30,31]. Therefore, we cannot know if the observed differences in HRQoL mattered to the patients. Finally, our pruritus questionnaire has not been validated and we observed frequencies of pruritus that were much higher than those in the other trials of HES in ICU patients as described above.

## Conclusions

Patients with severe sepsis assigned to resuscitation with HES 130/0.42 had worse self-perceived HRQoL at long-term follow-up than those who received Ringer's acetate, whereas there were no statistically significant differences in the occurrence of pruritus between the intervention groups. As these data add to the growing amount of evidence of worse outcome with HES in patients with severe sepsis, we recommend not using HES for these patients.

## Key messages

• The study showed that survivors of severe sepsis treated with hydroxyethyl starch scored significantly lower in SF-36 than those treated with Ringer's solution. The differences were seen in the mental component summary score, and the vitality, mental health, social function and bodily pain scales.

• The differences in frequency and severity of pruritus between the HES and Ringer's group were not statistically significant.

• Pruritus was very frequent in the sepsis survivors in the present trial.

## Abbreviations

6S: Scandinavian Starch for Severe Sepsis/Septic Shock; BP: bodily pain; GH: general health; HES: hydroxyethyl starch; HRQoL: health-related quality of life; ICU: intensive care unit; IQR: interquartile range; MCS: mental component summary; MDE: missing data estimation; MH: mental health; PCS: physical component summary; PF: physical functioning; RCI: response consistency index; RE: role emotional; RP: role physical; SF-36: short form-36; SF: social functioning; VAS: visual analog scale; VT: vitality.

## Competing interests

The 6S trial was funded by the Danish Strategic Research Council, the Danish Research Council and Rigshospitalet and supported by B Braun Medical AG. The Department of Intensive Care, Rigshospitalet receives research funds from Fresenius Kabi. Neither of these had any influence on the trial protocol or conduct nor data analyses or reporting.

## Authors' contributions

PW, NH and AP conceived and designed the study. PW and DB acquired the data. PW, NH, DB and AP analyzed and interpreted the data. PW drafted the manuscript. AP, NH, JBP, RW and DB critically revised the manuscript for important intellectual content. RW and PW provided administrative, technical, and material support. AP supervised the study. All of the above stated authors have read and approved the manuscript for publication. The 6S trial group was responsible for the original randomized controlled trial.

## Supplementary Material

Additional file 1 **6S inclusion and exclusion criteria, best/worst-case analyses and pruritus questionnaire**. Click here for file
