# Supplementary material for: Quality of life and pruritus in patients with severe sepsis resuscitated with hydroxyethyl starch long-term follow-up of a randomised trial
Source: Crit Care. 2013 Feb 25;17(2):R58. doi: 10.1186/cc12586 (PMC3672692; doi:10.1186/cc12586)
Supplement: Additional file 1 — 6S inclusion and exclusion criteria, best/worst-case analyses and pruritus questionnaire. [file cc12586-S1.DOC]

**Inclusion and exclusion criteria for the 6S trial**

The included patients were aged 18 years or above, needed fluid resuscitation in the ICU as judged by the clinicians and fulfilled the criteria for severe sepsis within the previous 24 hours [1][2].

Patients were excluded if they had previously been randomized, for medical reasons (renal replacement therapy, kidney or liver transplant within current admission, burn injury, intracranial bleeding within current admission or s-K^+^ > 6 mM within the last 6 hours), if they had received more than 1000 ml of synthetic colloid in the last 24 hours, if they were enrolled into another ICU trial of drugs with effects on circulation, renal function or coagulation or if consent could not be obtained.

.

**The best-worst scenario analyses**

In the Worst case scenario analysis, missing answers were imputed with the worst possible answer in the HES group and the best possible answer in the Ringer’s group. In the Best case scenario analysis, missing answers were imputed with the best possible answer in the HES group and the worst possible answer in the Ringer’s group.

|  | **Worst case scenario** | |  | | **Best case scenario** | |  |
| --- | --- | --- | --- | --- | --- | --- | --- |
|  | **HES N(95)** | **Ringer’s Group N(87)** |  | | **HES N(95)** | **Ringer’s Group N(87)** |  |
| **Scale** |  |  | **p-value** |  |  |  | **p-value** |
| **PCS** | 37 (29-45) | 42 (32-52) | 0.1 |  | 38 (30-48) | 40 (32-49) | 0.62 |
| **MCS** | 44 (33-54) | 54 (40-60) | 0.001 |  | 47 (37-55) | 50 (39-58) | 0.14 |
| **PF** | 50 (20-75) | 65 (30-85) | 0.07 |  | 50 (20-75) | 65 (30-80) | 0.45 |
| **RP** | 0 (0-75) | 25 (0-75) | 0.08 |  | 0 (0-100) | 0 (0-75) | 0.39 |
| **BP** | 52 (31-84) | 74 (42-100) | 0.006 |  | 60 (31-84) | 72 (41-100) | 0.06 |
| **GH** | 37 (25-62) | 52 (30-72) | 0.03 |  | 45 (30-67) | 47 (25-72) | 0.89 |
| **VT** | 45 (25-60) | 55 (35-75) | 0.001 |  | 45 (30-60) | 55 (35-75) | 0.06 |
| **SF** | 75 (38-100) | 88 (63-100) | 0.003 |  | 75 (38-100) | 75 (63-100) | 0.24 |
| **RE** | 33 (0-100) | 67 (0-100) | 0.02 |  | 67 (0-100) | 33 (0-100) | 0.71 |
| **MH** | 64 (52-80) | 80 (60-92) | 0.001 |  | 64 (52-84) | 80 (60-92) | 0.02 |

Median and interquartile (IQR) values for subscales and component summaries (PCS and MCS) of the HES and Ringer’s groups with the p-values calculated with Mann-Whitney U-test for independent samples. Data are without MDE imputation.

Abbreviations: PCS - physical component summary, MCS - mental component summary, PF - physical functioning, RP - role physical, BP- bodily pain, GH - general health, VT - vitality, SF - social functioning, RE - role emotional, MH - mental health.

The pruritus questionnaire translated from Danish

**Questionnaire about skin itching**

1. Have you experienced any itching today or yesterday? Yes______ No ______

Underneath we have drawn a scale from 0 to 10. No itching is 0, while 10 stand for the worst imaginable itching. If you answered yes to the question above, we want you to mark with a cross on the line, how bad the itching was.

0 1 2 3 4 5 6 7 8 9 10

No itching

Worst imaginable itching

2. Have you been bothered by itching in a shorter or longer period of time after your stay at the intensive care unit in 2009/2010/2011? Yes _____No_____

If yes, what kind of action did you take to relieve the itching (you may place more than one cross)?

None ______

I used an ointment/pills ______

I discussed the issue with my GP ______

I consulted the problem with a dermatologist_______

Other_____________________________________________________________________

**References**

1. Bone RC, Balk RA, Cerra FB, Dellinger RP, Fein AM, Knaus WA, Schein RM, Sibbald WJ: **Definitions for sepsis and organ failure and guidelines for the use of innovative therapies in sepsis. The ACCP/SCCM Consensus Conference Committee. American College of Chest Physicians/Society of Critical Care Medicine.** *Chest* 1992, **101:**1644-55

2. Perner A, Haase N, Guttormsen AB, Tenhunen J, Klemenzson G, Aneman A, Madsen KR, Moller MH, Elkjaer JM, Poulsen LM, Bendtsen A, Winding R, Steensen M, Berezowicz P, Søe-Jensen P, Bestle M, Strand K, Wiis J, White JO, Thornberg KJ, Quist L, Nielsen J, Andersen LH, Holst LB, Thormar K, Kjældgaard AL, Fabritius ML, Mondrup F, Pott FC, Møller TP, Winkel P, Wetterslev J; 6S Trial Group; Scandinavian Critical Care Trials Group: **Hydroxyethyl starch 130/0.42 versus Ringer's acetate in severe sepsis.** *N Engl J Med* 2012, **367:**124-134.
